# Bilateral ictal eye closure in focal epileptic seizures: SEEG retrospective observational assessment from a tertiary epilepsy center

**DOI:** 10.1002/epd2.70246

**Published:** 2026-04-13

**Authors:** Paola Vassallo, Christina Pourlou, Joana F. A. Oliveira, Davide Giampiccolo, Charlotte McLaughlin, Emma Torzillo, Umair Javaid Chaudhary, John S. Duncan, Josemir W. Sander, Fahmida Chowdhury, Beate Diehl

**Affiliations:** ^1^ Department of Clinical & Experimental Epilepsy UCL Queen Square Institute of Neurology London UK; ^2^ Chalfont Centre for Epilepsy London UK; ^3^ Stichting Epilepsie Instellingen Nederland (SEIN) Heemstede Netherlands; ^4^ Department of Neurology Leiden University Medical Centre Leiden Netherlands; ^5^ Department of Clinical Neurophysiology National Hospital Neurology and Neurosurgery London UK; ^6^ Victor Horsley Department of Neurosurgery National Hospital for Neurology and Neurosurgery London UK; ^7^ Department of Neurology West China Hospital, Sichuan University Chengdu China

**Keywords:** electroclinical correlation, epilepsy surgery, seizure semiology

## Abstract

Ictal eye closure is commonly associated with functional seizures but may also occur in epileptic seizures. We retrospectively reviewed 113 consecutive stereo‐EEG (SEEG) recordings from adults with drug‐resistant focal epilepsy (2015–2024) to identify prolonged bilateral eye closure during seizures. Nine individuals exhibited this through 17 seizures; eight seizures arose from sleep. Inter‐rater reliability was good (ICC = 0.72). The median age was 35 years, and the epilepsy duration was 25 years. Eye closure started mainly at EEG onset and persisted for approximately 50% of the seizure. Consciousness was impaired in seven seizures and preserved in three. Presumed epileptogenic zones were unilateral in eight people and uncertain in one, involving various cortical and subcortical areas. Seven of them were offered surgical resection or laser interstitial thermal therapy (LITT). Ictal eye closure showed no clear localizing or lateralizing value, likely due to widespread network involvement. It may reflect disruption of sensory, executive, or arousal systems. SEEG spatial sampling limitations could have affected the detection of involved areas. Further prospective studies are needed to clarify its clinical significance.

**Key points**

- Bilateral eye closure can occur in epileptic seizures but is rare and lacks localizing or lateralizing value.
- Three subnetworks emerged—limbic, posterior, and medial motor—with partial overlap supporting a distributed mechanism.
- Likely mechanisms include indirect motor pathway activation and interoceptive–sensorimotor integration.

## 1 INTRODUCTION

Closed eyes during a seizure have a high positive predictive value (PPV: 0.943) for functional seizures (FS),^1^ although it is not definitive.

Some studies reported eye closure in 5%–26% of epileptic seizures (ES) versus 34%–64% in FS episodes. Closure lasting more than half the episode duration had the highest predictive value for FS.^2^, ^3^

Several case reports have documented ictal eye closure in focal seizures arising from the frontal lobe,^4^ anterior cingulate cortex, and mesiotemporal structures^5^ and inferior temporal region, which is connected posteriorly with parieto‐occipital regions.^6^ However, anatomical structures involved at the time of the emergence of the eye closure have not been systematically assessed.

This retrospective observational assessment in adults undergoing stereo‐EEG (SEEG) to determine surgical candidacy aims to investigate the anatomical and electrophysiological correlates of bilateral ictal eye closure in epileptic seizures.

## 2 METHODS

Adults with drug‐resistant focal epilepsy who underwent SEEG (Micromed®) at the National Hospital for Neurology and Neurosurgery, London, between 2015 and 2024 were retrospectively reviewed. Consecutive SEEG reports were screened for ictal eye closure. Bilateral ictal eye closure was defined as complete eyelid closure lasting >5 s, including cases preceded by a blink or minimal residual scleral visibility. Tonic closure was defined as showing visible periorbital muscle activation; naturalistic closure was smoother. Ictal testing assessed alertness, verbal, motor responses, and keyword recall.^7^ Two independent raters (PV, CP) reviewed video‐SEEG to determine eye closure presence and duration, expressed as a percentage of total electrographic seizure time. Disagreements were resolved by a third rater (BD) or consensus.

Structures involved at the time of eye closure were identified via SEEG contacts showing the earliest ictal activity. Corresponding EEG patterns were documented. Eye closure was classified as early (within 5 s of EEG onset) or after propagation (>5 s).

Demographic, clinical, and SEEG data were collected for all patients, with histopathology recorded when surgery was performed. A multidisciplinary team assessed epilepsy type, semiology, neuropsychological and psychiatric findings, and imaging. Electrode implantation was guided by epilepsy phenotype and hypothesized epileptogenic zone (EZ). The presumed EZ was defined primarily on ictal SEEG, supported by concordant clinical and imaging data, and confidence in localization graded according to Delphi criteria.^8^

### 2.1 Statistical analysis

Quantitative variables are presented as medians and ranges. Inter‐rater reliability for eye‐closure duration used a two‐way random‐effects model with consistency type (intraclass correlation coefficient, ICC), using RStudio (Posit Software, PBC). Agreement was interpreted as poor (0.00–0.20), fair (0.21–0.40), moderate (0.41–0.60), good (0.61–0.80), or excellent (0.81–1.00).

## 3 RESULTS

One hundred and thirteen reports were reviewed. Bilateral eye closure during seizures was reported in 9 individuals, encompassing 17 spontaneous seizures.

The median monitoring duration was 9 days (range: 3–16). Seven individuals were male; one was left‐handed. The median age was 35 years (range: 25–48), the median age at epilepsy onset was eight years (range: 2–20), and the median epilepsy duration was 25 years (range: 10–46). Four had structural abnormalities (Table 1).

**TABLE 1:** Case summary.

N	Sex, age, handedness, age at epilepsy onset	Presumed epileptogenic zone	Seizure recorded (*N*)	Seizures with eyes closed	Eye closure occurrence and duration	MRI findings	Outcome	Schematic view of areas involved at the time of eye closure and EEG pattern
1	F, 30, R Epilepsy onset: 8 years	R insulo‐opercular Level of confidence: high	With eyes closed: 2 Total: 23	*Semiology*: 1. FIC with distal gestural automatisms > bilateral tonic (sleep) 2. FIC with L hand dystonic > distal gestural automatisms (wake)	At EEG onset 36%–45%	Nonlesional	LITT declined by patient. Follow‐up: 5 years	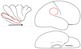 *EEG pattern*: Polyspikes of high amplitude rapidly evolving to LVFA 30 Hz. R parieto‐insular operculum
2	M, 25, R Epilepsy onset: 15 years	R temporo‐occipital Level of confidence: high	With eyes closed: 1 Total: 9 clinical, 70 subclinical (postimplantation hemorrhage)	*Semiology*: FIC with oroalimentary automatisms (wake)	Early after EEG onset, during spreading to posterior cingulum 50%	R parietal lobe gliosis, likely ischemic perinatal damage	Lesionectomy: 95% improvement in short postsurgical follow‐up Pathology: Focal cortical dysplasia type IIId, ILAE 2022 Follow‐up: 4 years	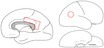 *EEG pattern*: Spike‐and‐wave activity evolving to LVFA 80 Hz. R temporo‐occipital junction and posterior cingulum, with late spread to the anterior hippocampus and precuneus
3	M, 39, R Epilepsy onset: 20 years	R posterior insula Level of confidence: high	With eyes closed: 1 Total: ~100	*Semiology*: FIC: Indescribable or somatosensory painful L face aura > complex motor distal automatisms (sleep)	At EEG onset 100%	Nonlesional	Awaiting LITT Follow‐up: 6 years	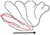 *EEG pattern*: LVFA 50 Hz R posterior insula
4	M, 44, R Epilepsy onset: 16 years	Uncertain if R temporal or multifocal Level of confidence: poor	With eyes closed: 2 Total: 7	*Semiology*: 1. FPC with cognitive phenomena > complex motor features oroalimentary automatisms (awake) 2. FIC with complex motor features oroalimentary automatisms (sleep)	At EEG onset and during spread toward cingulum cortex 17%–75%	Asymmetric amygdala R > L; nonspecific T2 hyperintense signal on the R	Medical management, not good surgical candidate Follow‐up: 6 years	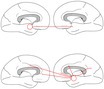 *EEG pattern*: Repetitive spikes or sharp waves, evolving to more diffuse slow. 1. Focal R hippocampal onset, early bilateral spread toward frontal and prefrontal cortex, TOJ, anterior and posterior cingulum, but the maximum remains in R hippocampus 2. Focal L hippocampal onset, subsequent R hippocampus involvement
5	M, 30, R Epilepsy onset: 8 years	Likely L orbitofrontal Level of confidence: very high	With eyes closed: 3 Total: 29 clinical ~100 subclinical	*Semiology*: 1. Focal and 2. FIC with autonomic phenomena (ictal tachycardia) > complex motor phenomena, gestural distal automatisms (sleep) 3. Subclinical (wake)	1 and 2. at EEG onset 3. during spread to hippocampus and amygdala, cause EEG onset less well defined 13%–50%	Nonlesional	L orbitofrontal resection: Engel 1A Pathology: focal cortical dysplasia type II a Follow‐up: 7 years	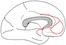 *EEG pattern*: Repetitive spikes, evolving to LVFA 1 and 2: L posterior orbitofrontal, with early involvement of mid‐cingulum 3. L posterior orbitofrontal, with spread to posterior hippocampus and amygdala
6	M, 35, R onset: 7 years	R temporal Level of confidence: moderate	With eyes closed: 1 Total: 59 Subclinical 54	*Semiology*: Focal with complex motor phenomena, oroalimentary automatisms (wake)	At EEG onset, until spread toward posterior insula 54%	R hippocampal sclerosis	Engel 4A following R temporal lobectomy Pathology: Hippocampal sclerosis type I Follow‐up: 9 years	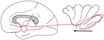 *EEG pattern*: Repetitive spikes or sharply contoured theta activity, evolving to LVFA. R hippocampus with rapid involvement of amygdala, and secondary spread to posterior insula, posterior cingulum and orbitofrontal cortex
7	F, 44, L onset: 14 years	Likely R anterior frontal and orbitofrontal Level of confidence: poor	With eyes closed: 2 Total: 11	*Semiology*: 1. FPC and 2. FIC with Complex motor phenomena: vocal > distal gestural automatisms > hyperkinetic behavior (wake)	1. After spreading to L parietal lobule, with L orbitofrontal still dysfunctional 2. after spreading to L orbitofrontal region and anterior insula and SMA 6%–22%	Nonlesional	R fronto‐orbital resection declined by patient. Follow‐up: 1 year*	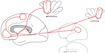 *EEG pattern*: Repetitive spikes, evolving to LVFA 30 Hz, then to diffuse slow. 1. R Orbitofrontal with rapid spread to L supramarginal gyrus, anterior Insula and L inferior parietal lobe 2. R Orbitofrontal with rapid spread to SMA and anterior insula
8	M, 33, R onset: 8 years	Likely L temporo‐occipital Level of confidence: moderate	With eyes closed: 4 Total: 7 Subclinical: 2	*Semiology*: 1–3. Focal with complex motor phenomena, oroalimentary and distal gestural automatisms (2/3 sleep, 1/3 wake) (not assessed) 4. subclinical (sleep)	At EEG onset 80%–100%	Nonlesional	Medical management and VNS, not good surgical candidate Follow‐up: 1 year*	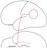 *EEG pattern*: 80 Hz gamma burst riding on infraslow at onset or 5–6 Hz spike and wave, transforming to gamma activity. L temporo‐parietal, angular gyrus, with spread to posterior hippocampus and amygdala
9	M, 48, R onset: 2 years	L temporal Level of confidence: very high	With eyes closed: 1 Total: 3	*Semiology*: FIC with complex motor phenomenal, oroalimentary automatisms, atonic head (wake)	Early after EEG onset, with sustained rhythmic activity in the L anterior hippocampus and involvement of posterior hippocampus 8%–12%	L hippocampal sclerosis and L parietal cavernoma	L temporal lobectomy: Engel 1A Pathology: L hippocampal sclerosis type I Follow‐up: 3 years*	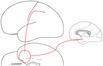 *EEG pattern*: Sharply‐contoured rhythmic activity in alpha range evolving to theta range. L anterior hippocampus and L parietal, later evolving to R parietal with a maximum remaining on the L

*Note*: Demographic characteristics, presumed epileptogenic zone with level of confidence according to the Delphi consensus, age at epilepsy onset, seizure type with eye closure, imaging findings, eye closure duration expressed as a percentage of whole seizure duration, outcome, graphic representation of epileptogenic zone, and EEG pattern in seizures with eye closure. Automatisms description: Oroalimentary: chewing, subtle mouth and jaw movements (Subject 1, 4, 8, 9) subtle lip and mouth movements (Subject 6). Distal gestural: subtle finger movements (Subjects 1, 2, 5). Other motor automatisms: repetitive face touching (Subject 1), fidgeting with nasal canula (Subject 2); intermittent irregular head movement (Subject 2 and 8); whole body movements, irregular (Subject 3 and Subject 7). Verbal: moaning (Subject 1); whistling (Subject 7); vocalization (Subject 7); simple word repetition (Subject 7). Follow‐up duration: * indicates nonactive follow‐up in Subjects 7, 8, 9, who were referred to local Epilepsy Center care.

Abbreviations: ECS, electrical cortical stimulation; LITT, laser interstitial thermal therapy; arrow in insula diagram indicates direction from posterior to anterior; LVFA, low‐voltage fast activity; TOJ, temporo‐occipital junction.

Inter‐rater agreement for eye closure duration was good (ICC = 0.72, 95% CI = 0.47, 0.86, *p* < 0.001).

Bilateral eye closure typically occurred at seizure onset, with a median time from SEEG onset of 2.5 s (range: 0–197). Eye closure occurred more than 10 s after SEEG onset in only five seizures. Eye closure persisted for an average of 53% of the total seizure duration (average eye closure duration 28 s, standard deviation 15, standard error 3, and average seizure duration 80 s, standard deviation 62, standard error 14).

Among the 17 seizures, eight occurred during sleep and nine during wakefulness. None of the seizures were induced by electro‐cortical stimulation (ECS).

Consciousness was lost at the time of eye closure in seven seizures, preserved in three, and not formally assessed in seven. Three people had bilateral eye closure in seizures with and without loss of consciousness.

Subtle positive motor signs were observed along with eye closure in sleep seizures such as a preceding blink (subjects 1 and 4), grimacing (4), or automatisms (5, 8 and 9). During wakefulness, eye closure co‐occurred with grimacing (subjects 3 and 4), automatisms (subjects 1, 2, 3, 8, 9), or behavioral changes while reading or using their phone (6, 9). In four cases (1, 3, 4, 7), eye closure was tonic, whereas in the remaining cases it appeared more naturalistic. Resistance to passive eye opening was not assessed. One subject (4) failed to open the eyes during the event, when asked; in most others, staff arrived at seizure offset or after eye opening. Some subjects reported emotional content related to seizures, such as sense of heaviness (1), feeling strange (4), or uncomfortable (3). In the other cases, facial expression was neutral.

**FIGURE 1:**
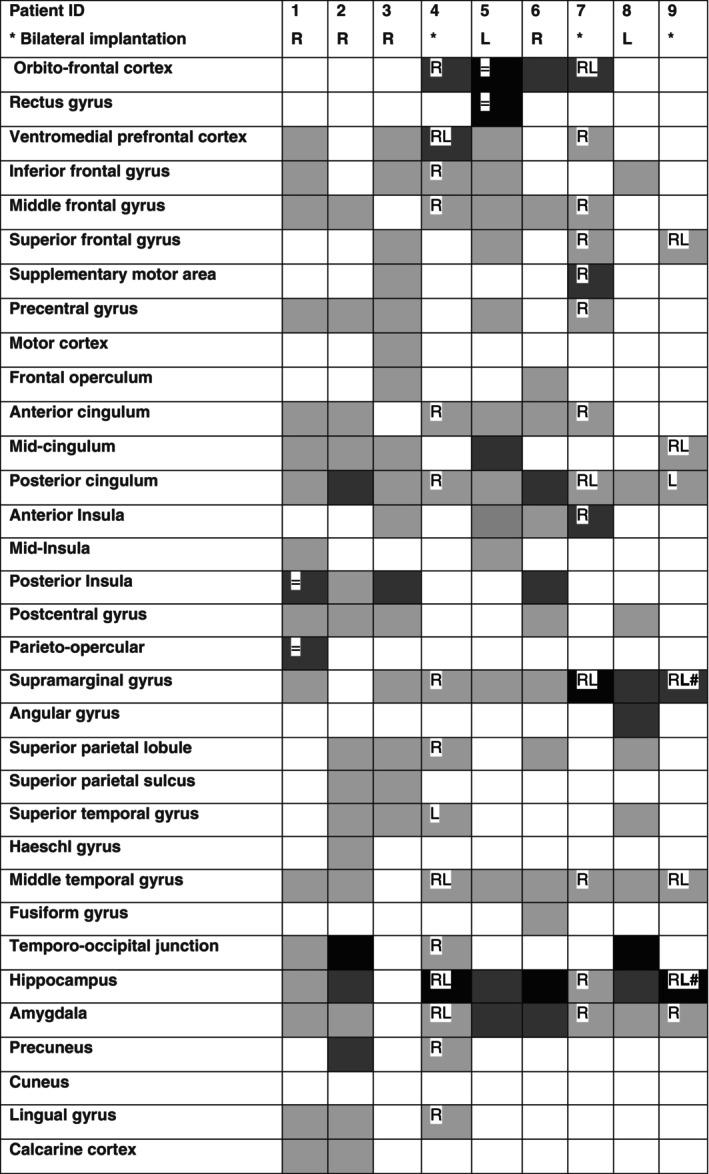
Regions explored through SEEG for each individual. Regions explored through SEEG for each individual. “*” indicates bilateral implantation. The light gray squares indicate anatomical regions explored and found not to be involved at the time of eye closure. The dark gray squares indicate anatomically explored areas found to be involved at the time of eye closure. The black squares indicate predominant regions based on earliest recruitment, highest amplitude, and sustained rhythmic ictal activity. The white squares indicate unexplored anatomical regions. R = right and L = left. Subject 9: Only L hippocampus and supramarginal gyrus were involved (#). (=) Indicates equal involvement.

The presumed epileptogenic zone was right hemispheric in five cases, left hemispheric in three, and uncertain in one. At the time of bilateral eye closure, predominant ictal involvement was hippocampal (4, 6, 9), posterior insular (1, 3), temporo‐occipital junction (2, 8), parietal (operculum 1, supramarginal gyrus 7) and orbitofrontal or prefrontal (5, 7). In several cases, additional regions were co‐active but showed later recruitment or lower amplitude (e.g., 2 TOJ to posterior cingulum and hippocampus; 8 TOJ to supramarginal and angular gyri, then hippocampus, 5 orbitofrontal to mid‐cingulum and hippocampus; 9 hippocampus to supramarginal gyrus), as summarized in Table 1 and Figure 1.

In four surgical patients, at least one structure involved at the time of bilateral ictal eye closure was resected (right parietal lesionectomy; left orbitofrontal, right temporal, and left temporal resections). Postoperative outcomes included two Engel 1A, one Engel 4A, and one with approximately 95% seizure reduction at short‐term follow‐up. One individual declined surgery. Two people were offered Laser interstitial thermal therapy (LITT); one declined, and the other is awaiting for the procedure. One had vagus‐nerve stimulator (VNS) implanted.

## 4 DISCUSSION

In our SEEG cohort, bilateral ictal eye closure lacked localizing or lateralizing value, suggesting a network‐driven behavior. Three subnetworks emerged: a limbic (orbitofrontal–mesiotemporal), a posterior (posterior insula–cingulum–parietal), and a medial motor (mid‐cingulum–SMA). Partial overlap supports a widespread mechanism.

The mid‐cingulum contributes to facial movements via frontal eye field connections, while anterior and posterior cingulum may modulate eye closure through emotional and sensory networks.^9^ The posterior cingulum has also been associated with eye closure and involuntary movements during ECS.^10^

Limbic regions (hippocampus and amygdala), despite lacking direct facial nucleus projections, can trigger ictal eye closure activating motor pathways, as supported by ECS studies^5^ and observations in subjects 2, 4, 5, 6, 8, and 9.

In some cases (1 and 3), eye closure may represent a behavioral response to pain or unpleasant sensory experiences. The insula and parietal operculum, involved in interoception and sensorimotor integration, likely contribute to this response via connections with the prefrontal and parietal cortices.^11^ Emotional or volitional facial paralysis from lesions in the operculum, cingulum, insula, or thalamus supports this interpretation.^12^

Most seizures with eye closure involved loss of awareness. This aligns with studies based on ECS, SEEG, and SPECT data,^5^ implicating limbic seizures in the disruption of the arousal network (involving the frontal cortex, brainstem, and thalamus), particularly in longstanding temporal lobe epilepsy.^13^ Loss of awareness in seizures from the frontal, parietal, or occipital lobes is less assessed.^14^

Eye closure was documented only in spontaneous seizures, which engage broader networks, but not in ECS, which targets discrete regions, suggesting sustained eye closure requires inter‐regional synchronization.

Many seizures occurred during sleep, where assessment of eye closure can be challenging. However, eye opening is typically expected at seizure onset,^15^ even during sleep. Persistent eye closure in this context may therefore represent an active ictal sign—either impaired eye opening mimicking apraxia, as seen in acute cortical lesions,^12^ or excessive eye closure activation—rather than a passive state. This interpretation is further supported by its association with other subtle positive motor signs (preceding blink, grimacing, oral and gestural automatisms).

Bilateral ictal eye closure, though rare, may reflect the epileptic network activation process and should not be dismissed as a standalone indicator of functional seizures. Instead, it should be considered within the broader context of seizure semiology to avoid misclassification and ensure accurate diagnosis.

Strengths of our assessment include SEEG sampling of relevant structures, evaluation of eye closure by independent raters, and a multidisciplinary, comprehensive review. Limitations include reliance on report‐based initial screening, small sample size, possible incomplete SEEG coverage, and lack of dedicated blink‐monitoring electrodes.

A relatively small number of patients achieved seizure freedom after surgery, limiting confidence in the proposed ictal network; however, seizure freedom occurred in cases with high confidence in EZ delineation.^8^ Moreover, SEEG provided precise localization, particularly in frontal and deep limbic regions prone to rapid spread, partially mitigating this limitation.

## 5 CONCLUSION

Bilateral ictal eye closure is rare during epileptic seizures and did not show localizing or lateralizing value in our SEEG cohort. It likely reflects a network dysfunction rather than a specific localization. The interaction and modulation of the involved cortical components remain unclear. Further work, combining semiology from SEEG with connectivity analyses in larger samples, is needed.

## AUTHOR CONTRIBUTIONS

**PV:** conceptualization; clinical data curation and analysis; writing—original draft preparation; editing. **CP:** clinical data curation and analysis; review and editing. **JFAO:** clinical data curation and analysis; technical contribution; review and editing. **DG:** clinical data curation and analysis; review. **CML:** clinical data curation; technical contribution. **ET:** clinical data curation and analysis; review. **UJC:** clinical data curation and analysis; review. **JSD:** clinical data curation; review and editing. **JWS:** review and editing. **FC**: clinical data curation and analysis; review. **BD**: conceptualization, supervision; clinical data curation and analysis; review and editing. All authors have read and agreed to the published version of the manuscript.

## CONFLICT OF INTEREST STATEMENT

The authors have no conflict of interest related to this work.

**Test yourself**

1. Approximately what proportion of epileptic seizures (ES) show ictal eye closure according to previous studies?
  - 1%–4%
  - 5%–26%
  - 34%–64%
2. Which structures are involved in supranuclear control of blinking?
  - Primary and secondary cortices, cingulate cortex, cerebellum, limbic structures
  - Basal ganglia, medulla, occipital cortex
  - Brainstem only
3. Bilateral ictal eye closure in seizures typically indicates:
  - Localizing value to one hemisphere
  - Lateralizing value to the frontal lobe
  - Network‐driven behavior across multiple regions

Answers may be found in the *Supporting information*.

## Supporting information


Data S1.

## Data Availability

The data supporting our findings are available on reasonable request to the corresponding author. The data are not publicly available due to privacy or ethical restrictions.
