# Supplementary material for: Bilateral ictal eye closure in focal epileptic seizures: SEEG retrospective observational assessment from a tertiary epilepsy center
Source: Epileptic Disord. 2026 Apr 13;28(3):868–77. doi: 10.1002/epd2.70246 (PMC13276683; doi:10.1002/epd2.70246)
Supplement: Supplementary file 1 — Data S1. [file EPD2-28-868-s001.docx]

1. Correct answer: B) 5%–26%.

2. Correct answer: A) Primary and secondary cortices, cingulate cortex, cerebellum, limbic structures.

3. Correct answer: C) Network-driven behavior across multiple regions.
